# Elevated IgG4 in patient circulation is associated with the risk of disease progression in melanoma

**DOI:** 10.1080/2162402X.2015.1032492

**Published:** 2015-06-03

**Authors:** Panagiotis Karagiannis, Federica Villanova, Debra H Josephs, Isabel Correa, Mieke Van Hemelrijck, Carl Hobbs, Louise Saul, Isioma U Egbuniwe, Isabella Tosi, Kristina M Ilieva, Emma Kent, Eduardo Calonje, Mark Harries, Ian Fentiman, Joyce Taylor-Papadimitriou, Joy Burchell, James F Spicer, Katie E Lacy, Frank O Nestle, Sophia N Karagiannis

**Affiliations:** 1 St. John's Institute of Dermatology; Division of Genetics and Molecular Medicine; Faculty of Life Sciences and Medicine; King's College London & NIHR Biomedical Research Centre at Guy's and St. Thomas' Hospitals and King's College London; Guy's Hospital; King's College London; London, UK; 2 University Hospital of Hamburg Eppendorf; Department of Oncology; Hematology and Stem Cell Transplantation; Hamburg, Germany; 3 Department of Research Oncology; Division of Cancer Studies; Faculty of Life Sciences and Medicine; King's College London; Guy's Hospital; London, UK; 4 King's College London; Faculty of Life Sciences and Medicine; Division of Cancer Studies; Cancer Epidemiology Group; Guy's Hospital; London, UK; 5 Wolfson Center for Age-Related Diseases; King's College London; London, UK; 6 Breakthrough Breast Cancer Research Unit; Department of Research Oncology; Guy's Hospital; King's College London School of Medicine; London, United Kingdom; 7 Skin Tumor Unit; St. John's Institute of Dermatology; Guy's Hospital, King's College London and Guy's and St Thomas' NHS Trust; London, UK; 8 Clinical Oncology; Guy's and St. Thomas's NHS Foundation Trust, London, UK

**Keywords:** B cells, biomarker, cancer inflammation, humoral response, immunomodulation, immunomonitoring, immunosuppression, IgG4, melanoma, prognosis

## Abstract

Emerging evidence suggests pathological and immunoregulatory functions for IgG4 antibodies and IgG4^+^ B cells in inflammatory diseases and malignancies. We previously reported that IgG4 antibodies restrict activation of immune effector cell functions and impair humoral responses in melanoma. Here, we investigate IgG4 as a predictor of risk for disease progression in a study of human sera (n = 271: 167 melanoma patients; 104 healthy volunteers) and peripheral blood B cells (n = 71: 47 melanoma patients; 24 healthy volunteers). IgG4 (IgG4/IgG_total_) serum levels were elevated in melanoma. High relative IgG4 levels negatively correlated with progression-free survival (PFS) and overall survival. In early stage (I–II) disease, serum IgG4 was independently negatively prognostic for progression-free survival, as was elevation of IgG4^+^ circulating B cells (CD45^+^CD22^+^CD19^+^CD3^−^CD14^−^). In human tissues (n = 256; 108 cutaneous melanomas; 56 involved lymph nodes; 60 distant metastases; 32 normal skin samples) IgG4^+^ cell infiltrates were found in 42.6% of melanomas, 21.4% of involved lymph nodes and 30% of metastases, suggesting inflammatory conditions that favor IgG4 at the peripheral and local levels. Consistent with emerging evidence for an immunosuppressive role for IgG4, these findings indicate association of elevated IgG4 with disease progression and less favorable clinical outcomes. Characterizing immunoglobulin and other humoral immune profiles in melanoma might identify valuable prognostic tools for patient stratification and in the future lead to more effective treatments less prone to tumor-induced blockade mechanisms.

## Abbreviations

LDH: lactate dehydrogenase
CTC: circulating tumor cell
SD: stable disease
PD: progressive disease
Treg: regulatory T cell
CRP: C-reactive protein
WBC: white blood cell count
ROC: receiver operating characteristic
AUC: area under the curve
PFS: progression-free survival (PFS)
OS: overall survival
HR: hazard ratio
CI: confidence interval
SNB: sentinel lymph node biopsy
TMA: tissue microarray
PBMC: peripheral blood mononuclear cells

## Introduction

Malignant melanoma remains a potentially lethal skin cancer despite emerging targeted therapies.^1-4^ Approximately 20–50% of individuals diagnosed with early stage disease will develop metastases, yet current serum and histopathological evaluators are not linked to disease mechanisms and thus may not accurately predict the risk of disease recurrence.^4^ Prognosis largely relies on histological evaluation of the primary lesion when available, including Breslow thickness, ulceration and mitotic rate, together with assessments of nodal involvement, all of which require invasive surgical interventions.^5^ While sentinel node biopsy has been demonstrated to provide valuable prognostic information, around 80% of patients have a negative test but may develop long-term complications from surgery such as lymphoedema.^6-8^ Hence, alternative non-invasive indicators linked to a biological mechanism, such as induction of immune suppression by melanoma cells, would be highly valuable prognostic tools especially in early disease.

The only serological biomarker for melanoma recommended in the American Joint Committee on Cancer guidelines is lactate dehydrogenase (LDH).^4^ Elevated serum LDH levels are found in patients with progressive disease manifesting at later stages of melanoma. High serum LDH levels are indicative of active cell necrosis associated with high tumor burden and are linked to poor clinical responses to treatments.^4,9,10^ Serum biomarker candidates such as S-100B demonstrate high sensitivity and specificity in advanced disease, but have not yet been applied in routine clinical practice partly due to large variations among patient samples.^11^ Detecting circulating tumor cells (CTCs) may also be indicative of active disease in patients with advancing and metastatic tumors, signifying a worse clinical prognosis; however large inter- and intra-patient variability in cancer cell antigen expression and large variations in CTC counts between blood draws represent significant limitations.^12-14^ Regardless, markers that are easily monitored (such as those in serum) and that may be directly linked to early disease pathogenesis or progression are desirable.^15^

Components of humoral immunity –including circulating antibodies– are emerging as biomarkers for autoimmune, inflammatory or allergic conditions and malignant disease. Monitoring antibodies in disease may provide 2 advantages: a) responses may occur at early points of pathogenesis indicating likely future disease onset or recurrence; and b) B cell differentiation, class-switching and antibody production are influenced by antigen recognition, or altered in response to inflammatory signals, with the resulting antibodies constituting a convenient readout. Protein array platforms like ‘immunosignature’ are now being developed to monitor circulating antibodies, and applications in Alzheimer's disease and in cancer are emerging.^16,17^

Consistent with the notion that components of the humoral response could be associated with malignancy, early data indicated that IgG4 antibody subclass serum levels are dysregulated in patients with melanoma.^18^ Pathogenic roles of IgG4 in inflammatory diseases and also in pancreatic cancers, extrahepatic cholangiocarcinomas, squamous cell carcinomas and melanomas have also been described. Pathological features include elevated or dysregulated IgG4 serum levels, tissue-resident IgG4^+^ immune cell infiltrates and associations with regulatory elements such as regulatory T cells (Tregs).^18-20^ Additionally, correlations with inadequate immune responses to vaccines and with immune tolerance to allergen exposure following successful allergen immunotherapies have been reported.

We previously reported that despite the presence of a tumor-reactive mature humoral compartment in patients, production of IgG4 subclass antibodies may be favored in melanoma and that IgG4 may contribute to defective antitumor immune responses.^21-23^ These findings mandate a closer examination of the clinical significance of this immunomodulatory antibody subclass, particularly its association with disease progression. Here, in a cohort of patients and healthy volunteers, we sought to examine the levels of circulating IgG4 and IgG4^+^ B cells and determine if there is an association with disease progression in melanoma.

## Results

### Elevated IgG4 serum levels in patients predict the risk of disease progression and survival

**Figure 1.**
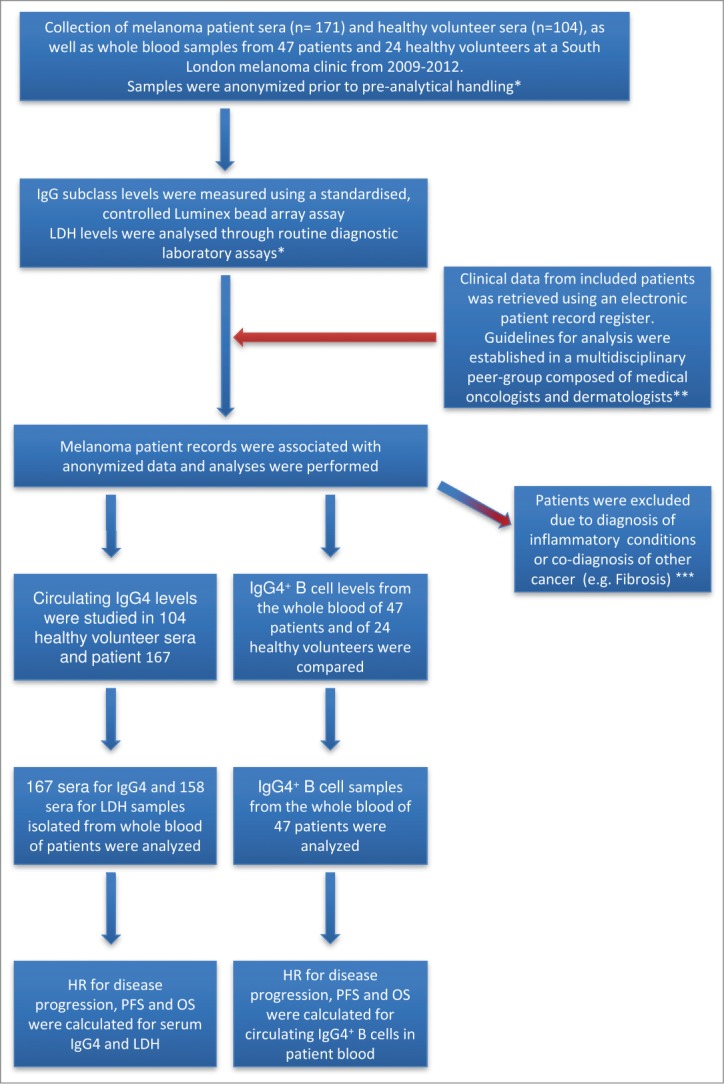
Experimental study design, collection and processing of clinical samples. Cohorts of 171 melanoma patients and 104 healthy volunteers were identified for evaluations of IgG4 and lactate dehydrogenase (LDH). IgG subclass levels were measured using a standardized Luminex bead array assay (n = 171) and in the same samples, LDH levels were analyzed through a diagnostic laboratory protocol (n = 158). *Researchers working on this study were blinded to prevent bias; **Two independent medical professionals, not involved in the quantification of IgG4 or LDH, evaluated patient information; ***Patients with co-morbidities that may influence IgG4 levels were excluded from the analysis.

**Figure 2.**
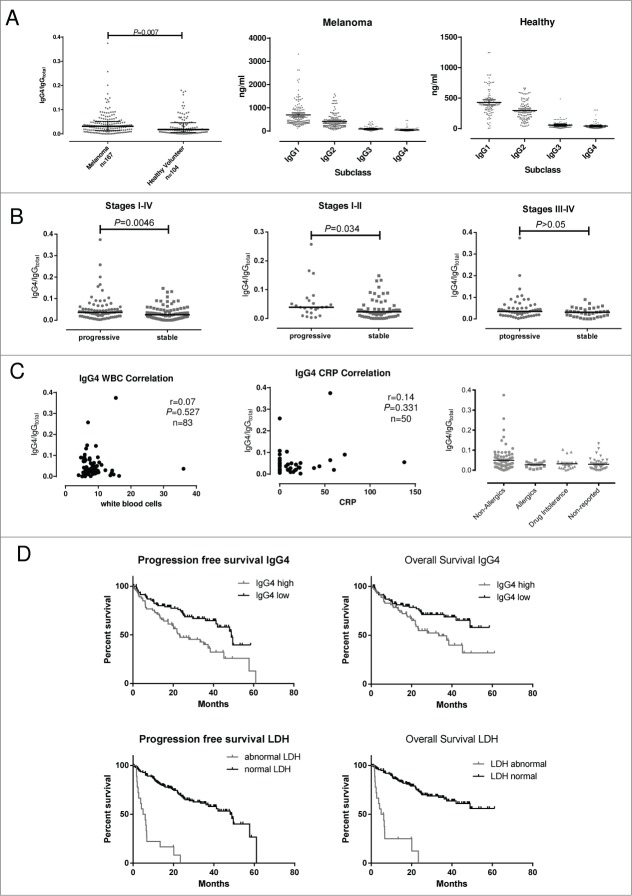
For figure legend, see next page.

In order to investigate circulating IgG4 in malignant disease, we analyzed sera from 167 patients with melanoma and from 104 healthy volunteers. Patient baseline characteristics are described in **Table S1** and the study design is described in **Figure 1**. Significantly elevated serum IgG4 levels (IgG4/IgG_total_) were detected in melanoma patients (median 0.031; 95%[CI] 0.036–0.051) as compared to healthy controls (median 0.017; 95%[CI] 0.026–0.042; *P* = 0.007; **Figure 2A, left**). The absolute concentrations of IgG subclasses for each cohort are shown in **Figure 2A** (center and right). In order to study the association between the levels of IgG4 and disease progression, clinical data from 167 patient sera were analyzed by stratifying patients into those with stable disease (SD) and those with progressive disease (PD) during the study period. In early disease (Stages I–II) patients with SD during the study period displayed significantly lower serum levels of IgG4 (IgG4/IgG_total_; median 0.023; 95%[CI] 0.024–0.044) as compared to patients who developed PD (median 0.039; 95%[CI] 0.030–0.0805; *P* = 0.034; **Figure 2B**). For all disease stages (Stages I–IV), patients with SD displayed significantly lower serum levels of IgG4 (IgG4/IgG_total_; median 0.025; 95%[CI] 0.026–0.039) than patients who developed PD (median 0.037; 95%[CI] 0.038–0.065; *P* = 0.0046; **Figure 2B**). To exclude any effects introduced by infection or inflammation on IgG4 levels in this study, we further examined potential correlations between IgG4 with C-reactive protein (CRP) and white blood cell count (WBC). Neither WBC (r = 0.07: *P* = 0.527: n = 83) nor CRP (r = 0.14: *P* = 0.331: n = 50) correlated with IgG4 levels in this study cohort (**Fig. 2C**). Furthermore, in our patient cohort, the levels of serum IgG4 (IgG4/IgG_total_) were not significantly different between non-allergic individuals and those with a history of allergies or patients with known drug intolerances (**Fig. 2C**).

Receiver operating characteristic (ROC) and area under the curve (AUC) analyses ^24^ indicated that elevated IgG4 could predict the risk of melanoma progression in local disease (mean AUC:0.65, *P* = 0.034 for Stages I–II) and overall (AUC:0.62, *P* = 0.005 for Stages I–IV) (**Figure S1A**). These findings suggest a prognostic value for IgG4 serum levels.

Since lactate dehydrogenase (LDH) is so far the only clinically-used serum biomarker used to predict disease progression,^4^ the potential of patient sera IgG4 and LDH levels to predict clinical outcomes were analyzed according to patient progression-free survival (PFS) and overall survival (OS). Based on ROC analysis, a threshold of 0.0345 for IgG4 was calculated via Youden's Index ^25^ (n = 91 in IgG4^low^, <0.034; n = 76 in IgG4^high^, ≥0.034). Using this cutoff point, IgG4 had a sensitivity of 57.14% (95%[CI] 45.35%–68.37%) and a specificity of 68.89% (95%[CI] 58.26%–78.23%), giving a likelihood ratio of 1.73.

For analysis of LDH, samples were segregated into groups according to normal (240–480 mmol/L, n = 140) and abnormal (>480 mmol/L, n = 18) sera concentrations. The thresholds for IgG4 and LDH were subsequently used to analyzed patient serum IgG4 and LDH (hazard ratios, HR) for PFS and OS using multivariate analysis (**Fig. 2D**). Kaplan-Meier curve evaluations revealed that IgG4^high^ group displayed statistically significantly lower OS (HR 95%[CI] 1.90 (1.17–3.29); log-rank *P* = 0.0116) and lower PFS (HR 95%[CI] 2.01 (1.34–3.35); log-rank *P* = 0.0016) as compared to the IgG4^low^ group (**Fig. 2D, top panel**). Patients with abnormal LDH levels had lower OS (HR 95%[CI] 7.71 (56.68–723.3); log-rank *P* < 0.0001) and lower PFS (HR 95%[CI] 6.93 (43.34–427.5); log-rank *P* < 0.0001) as compared to those with normal LDH (**Fig. 2D, bottom panel**). These data support the value of both serum LDH and IgG4 in predicting overall disease outcomes in melanoma. Moreover, these findings are consistent with an association between immune evasion mechanisms, such as those that promote IgG4, with subsequent disease progression.

Age, sex and, when appropriate, stage-adjusted Hazard Ratios (HR) and 95% Confidence Intervals (CI) were calculated for the risk of progression-free survival. The adjusted HR for IgG4 was 1.23 (95%[CI] 0.76–1.98) versus 3.64 (95%[CI] 1.85–7.19) for LDH in Stage I–IV disease. In Stages III–IV, IgG4 had an adjusted HR of 0.99 (95%[CI] 0.55–1.77) whereas LDH had an adjusted HR of 5.47 (95%[CI] 2.47–10.92). However, in the sera of patients with Stage I–II melanoma, IgG4 had an adjusted HR of 2.46 (95%[CI] 1.01–6.02) for predicting progressive disease, whereas serum LDH had an adjusted HR of 12.29 (95%[CI] 1.18–128.00) (**Table 1**). In summary, these data confirm the prognostic value of LDH and indicate that IgG4 serum levels provide significant prognostic information in early stages (i.e., Stage I–II) of melanoma.

**Table 1.** Clinical characteristics of the patient cohort, hazard ratio prediction calculations and correlations with serum IgG4 and LDH levels. Patients were categorized into 2 groups: those with low IgG4 levels (IgG4^low^: IgG4/IgG_total_ <0.034; n = 91) and those with high IgG4 levels (IgG4^high^ IgG4/IgG_total_ ≥0.034; n = 76), based on ROC analysis calculated via the Youden's Index. For LDH, samples were segregated into normal (240–480 mmol/L, n = 140) and abnormal (>480 mmol/L, n=18) concentrations. IgG4 predicted the risk of disease progression overall (combined Stages I–IV) as well as in local disease (Stages I–II). In contrast, LDH had a significant HR to predict the risk of disease progression overall (combined Stages I–IV). ^ˆ:^: Age, sex, and when appropriate stage adjusted Hazard Ratios (HR) and 95% Confidence Intervals (CI) for risk of disease progression

Patient Cohort	IgG4 <0.034 (n=91)	IgG4 ≥0.034 (n=76)
Mean age ± SD^*^	57 ± 18	64 ± 16
Sex		
Male (%)	39 (42.85)	45 (59.21)
Female (%)	52 (57.15)	31 (40.79)
Mean Breslow ± SD	2.53 ± 2.24	3.36 ± 2.32
Disease Stage (%)		
I	28 (30.77)	7 (9.21)
II	18 (19.78)	26 (34.21)
III	24 (26.37)	24 (31.58)
IV	21 (23.08)	19 (25.00)
Ulceration (%)		
None	51 (56.04)	30 (39.47)
Present	17 (18.68)	30 (39.47)
Unknown HR^ˆ:^ (95%CI) Stage I–II Stage III–IV Stage I–IV	10 (25.28)	16 (21.06) 2.46 (1.01–6.02) 0.99 (0.55–1.77) 1.23 (0.76–1.98)
Patient Cohort	LDH (240–480) (n=140)	LDH >480 (n=18)
Mean age ± SD	59 ± 18	64 ± 15
Sex		
Male (%)	70 (50.00)	11 (58.82)
Female (%)	70 (50.00)	7 (41.18)
Mean Breslow ± SD	2.87 ± 2.38	3.01 ± 1.81
Disease Stage (%)		
I	33 (23.57)	0 (0)
II	41 (29.29)	1 (5.56)
III	43 (30.71)	2 (11.11)
IV	23 (16.43)	15 (83.33)
Ulceration (%)		
None	74 (55.04)	5 (27.78)
Present	38 (25.58)	7 (38.89)
Unknown HR^ˆ:^ (95%CI) Stage I–II Stage III–IV Stage I–IV	28 (19.38)	6 (33.33) 12.29 (1.18–128.00) 5.47 (2.74–10.92) 3.64 (1.85–7.19)

* SD: standard deviation.

ˆ: Age, sex and, when appropriate, stage adjusted Hazard Ratio (HR) and 95% Confidence Intervals (CI) for risk of disease progression.

We next evaluated the prognostic potential of the combination of LDH and IgG4. When combined, these readouts had a higher prognostic value (AUC:0.67; *P* = 0.0002) than LDH (AUC:0.65; *P* = 0.0017) or IgG4 (AUC:0.60; *P* = 0.0227) individually (**Fig. S1B**).

### Elevated levels of peripheral blood IgG4^**+**^ B cells predict the risk of disease progression in Stage I–II melanoma

**Figure 3.**
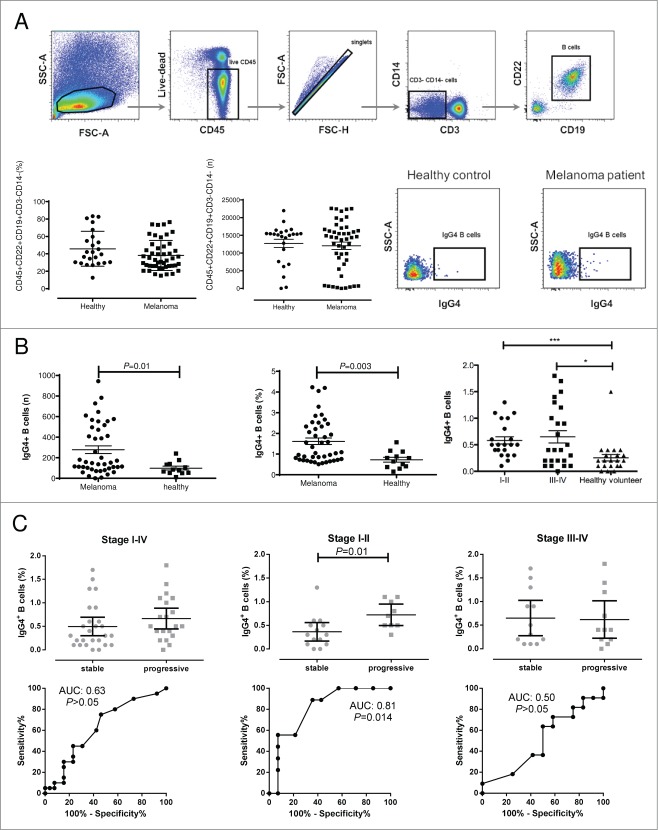
For figure legend, see next page.**Figure 3 (See previous page).** Increased frequencies of peripheral blood IgG4+ B cells from melanoma patients compared to healthy volunteers. (**A**) Representative cytofluorimetric dot plots and flow cytometry gating strategy for evaluation of the circulating IgG4^+^ B cell compartment. Lymphoid cells were gated according to their FSC-A and SSC-A properties and viable CD45^+^ were selected and cell doublets excluded using FSC-A and FSC-H dot plots. CD3^−^CD14^−^ cells were selected and B cells identified as CD19^+^CD22^+^ cells (top panel). Although the overall number of circulating B cells did not differ significantly between melanoma patients and healthy volunteer samples (lower panel, left for % of total PBMCs and numbers of B cell events), the IgG4^+^ cells were selected from the CD45^+^CD22^+^CD19^+^CD3^−^CD14^−^ cell compartment. Representative dot plots depicting IgG4^+^ (CD45^+^CD22^+^CD19^+^CD3^−^CD14^−^) peripheral B cells from PBMCs of a healthy volunteer (middle) and of a melanoma patient (right) (lower panel). (**B**) Left and Middle: The number (left) and frequency (middle) of the IgG4^+^ peripheral B cell compartment (based on counted CD45^+^CD22^+^CD19^+^CD3^−^CD14^−^ cells) of 24 healthy volunteer and 47 melanoma patient samples showed statistically significantly higher levels of IgG4^+^ B cells in the patient group (Mann-Whitney-U-test; *P* = 0.01; *P* = 0.003); Right: IgG4^+^ B cell frequencies differed significantly between Stages I–II (n = 24; *** *P* <0.001) or Stage III–IV (n = 23; * *P*<0.05) *vs.* healthy volunteers (n = 24). Statistical analysis was performed by Kruskal-Wallis one-way analysis of variance with post-hoc Dunn's test; lines represent medians and error bars indicate interquartile range. (**C**) Frequency of circulating IgG4^+^ B cells from patients with local (Stages I–II) or metastatic (Stages III–IV) melanoma and correlation with risk of disease progression. Statistical analysis was performed by Mann-Whitney-U-test; lines represent medians and error bars indicate interquartile range. Corresponding ROC analyses are depicted directly underneath each column graph. In the Stage I–II patient cohort, patients with stable disease had a significantly lower frequency of IgG4^+^ B cells *vs.* patients with progressive disease (*P = 0.014). For this cohort, a median AUC of 0.81 was calculated by ROC analysis.

Since serum IgG4 levels were predictive of the risk of disease progression in earlier stages (Stages I–II) of melanoma, we further examined patient and healthy volunteer peripheral blood for the presence of corresponding circulating IgG4^+^ B cells. The frequencies of circulating IgG4^+^ peripheral blood B cells (IgG4^+^CD45^+^CD22^+^CD19^+^CD3^−^CD14^−^) in melanoma patients with melanoma compared to those from healthy volunteers were determined by flow cytometric analyses (**Fig. 3A**). While there were no significant differences in the numbers of circulating B cells (CD45^+^CD22^+^CD19^+^CD3^−^CD14^−^) in the blood of patients with melanoma compared with healthy volunteers (**Fig. 3A**), the proportion of IgG4^+^ cells in the peripheral blood B cell compartment (CD45^+^CD22^+^CD19^+^CD3^−^ CD14^−^) of melanoma patients was significantly elevated (n = 47; median 0.50; 95%[CI] 0.47–0.73) compared with that of healthy volunteers' (n = 24; median 0.2; 95%[CI] 0.13–0.37; Mann-Whitney-U-test; *P* = 0.003) (**Fig. 3B**). Furthermore, significantly (Kruskal–Wallis one-way analysis of variance with post-hoc Dunn's test) higher frequencies of circulating IgG4^+^ B cells were detected in the blood of patients with Stage I–II (median 0.50; 95%[CI] 0.44–0.72; *P* < 0.001; n = 24) and Stage III–IV (median 0.40; 95%[CI] 0.41–0.89; *P* < 0.05; n=23) disease than those of healthy volunteers (median 0.2; 95%[CI] 0.13–0.37; n = 24) (**Fig. 3B, right**).

The frequencies of circulating IgG4^+^ B cells from patients with melanoma diagnosed at different disease stages were further analyzed to examine whether they could predict the risk of disease progression (Mann-Whitney-U-test; **Fig. 3C**). In the Stage I–II patient cohort, patients with stable disease had a statistically significantly lower frequency of IgG4^+^ B cells than patients who developed progressive disease (Stages I–II; AUC:0.81; *P* = 0.014; median of SD 0.30; 95%[CI] 0.17–0.56 *vs.* median of PD 0.70; 95%[CI] 0.50–0.95). There were no statistically significant differences between SD and PD patient groups in Stages III–IV (AUC:0.50; *P* > 0.05; median of SD 0.40; 95%[CI] 0.26–1.03 *vs.* median of PD 0.40; 95%[CI] 0.22–1.02) or in Stages I–IV (AUC:0.63; *P* > 0.05; median of SD 0.30; 95%[CI] 0.30–0.69 *vs.* median of PD 0.50; 95%[CI] 0.44–0.88).

Thus, similar to the prognostic value of elevated serum IgG4 levels in early stage (Stage I–II) melanoma, these data suggest that the IgG4^+^ B cell compartment is elevated in the circulation of patients as compared to healthy volunteers, and further, that elevated IgG4^+^ B cell frequencies are predictive of the risk of disease progression in Stage I–II melanoma.

### IgG4^**+**^ cell infiltration in cutaneous melanomas of different thickness, disease stage and in distant metastases

**Figure 4.**
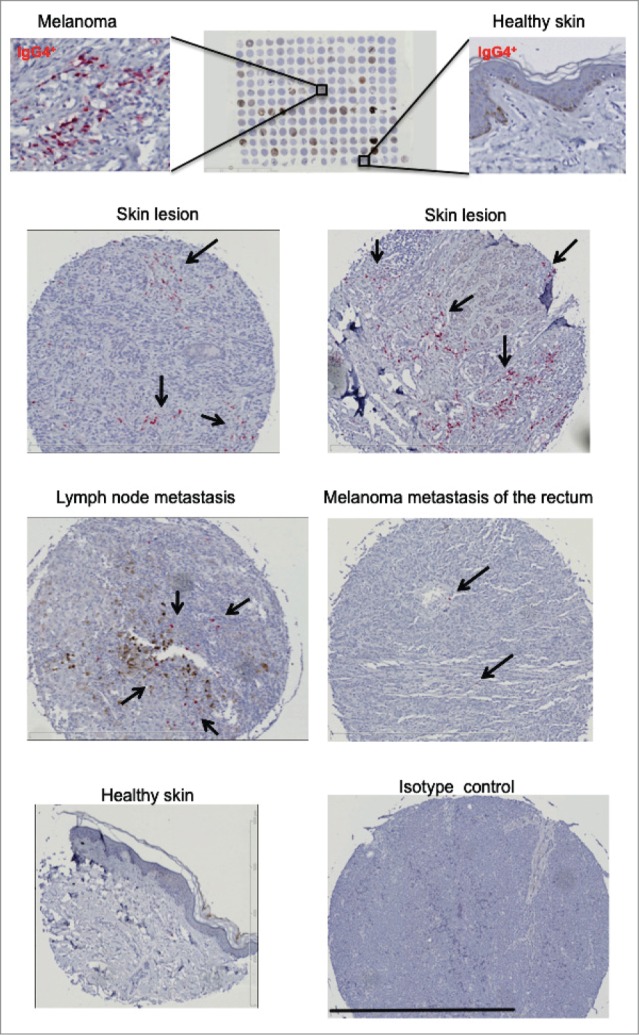
IgG4+ cell infiltrates are detected in melanoma skin tumors. A tissue microarray (TMA; BioMax, n = 256) consisting of melanoma skin tumors (n = 108), melanoma lymph nodes metastases (n = 56), distant organ metastases (n = 60) and healthy skin (n = 32) specimens (top panel) was examined for the presence of IgG4 by immunohistochemistry. IgG4 positive infiltrates were detected by alkaline phosphatase (in red, selected areas shown by black arrows) and sections were counterstained in hematoxylin (in blue). Representative images of IgG4 immunohistochemical staining for IgG4^+^ infiltration revealed positive staining in skin lesions (top and second panels), lymph node and distant metastases (third panel), while staining was less frequent in healthy skin (bottom panel). Black bar represents 800 µm (third bottom right panel).

**Figure 5.**
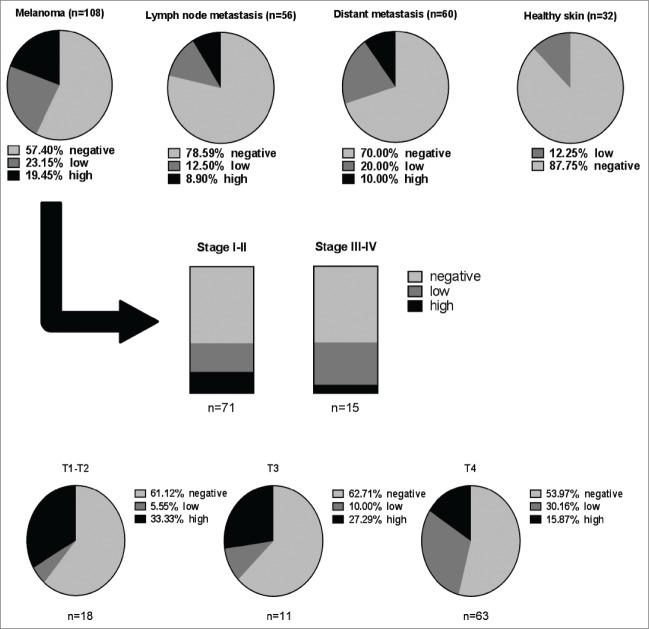
IgG4+ cell infiltration is higher in melanoma skin tumors, melanoma lymph nodes metastases, and distant organ metastases, compared to healthy skin. Top panel: Evaluation of tissue microarray (TMA) sections for frequency of IgG4^+^ cell infiltration in melanoma tissues in skin, lymph node metastases and distant metastases *vs.* healthy skin (Pie charts represent % of total numbers). Middle panel: In skin lesions (n = 108), IgG4^+^ infiltration is found in early (Stage I–II) and metastatic (Stage III–IV) disease. Lower panel: In melanoma skin lesions, IgG4^+^ cell infiltration is demonstrated across different melanoma skin tumor thicknesses (T1-T4). Analyses were performed by 2 independent researchers to identify the density of infiltration per high power field using the following criteria: “negative” = 0% infiltration; “low” < 25% infiltration; “high” > 25% infiltration.

In order to evaluate whether elevated serum IgG4 and circulating IgG4^+^ B cells in melanoma were also reflected by tumor IgG4^+^ cell infiltration, we performed immunohistochemical analyses of tissue microarrays (n = 256) for the presence of tissue-resident IgG4^+^ cells. IgG4^+^ cell infiltration was detected in a proportion of melanoma tumors. Levels of infiltration were found in melanoma skin lesions (n = 108), melanoma lymph node metastases (n = 56) and distant organ metastases (n = 60) but only low levels of infiltration were found in healthy skin (n=32) specimens (examples in **Fig. 4**). IgG4 expression was detected in 42.60% (19.45% high positivity) of melanoma skin lesions, 21.40% (8.90% high positivity) of lymph node metastases and 30% (10% high positivity) of distant metastases. Low-grade positivity was found in a smaller proportion (12.25%) of healthy skin specimens (**Fig. 5**).

Similarly, IgG4^+^ cell infiltrates were observed in local and metastatic disease [(Stage I–II: 39.43% (16.90% high; 22.53% low); Stage III–IV: 40% (6.67% high; 33.33% low)]. IgG4^+^ cell infiltrates were also found in skin tumor lesions of different thicknesses [(T1-T2: 38.88% (33.33% high; 5.55% low); T3: 37.29% (27.29% high; 10% low); T4: 46.03% (15.87% high; 15.87% low)] (**Fig. 5**). These findings may signify that IgG4-attributed immunosuppressive functions may occur throughout malignant disease, irrespective of skin tumor thickness.

Taken together, these data further support the value of circulating IgG4 and IgG4^+^ B cells as negative prognostic indicators in melanoma. Furthermore, IgG4^+^ infiltration in melanoma lesions may indicate that immunomodulatory mechanisms favoring IgG4-biased inflammation may also be relevant in tumor microenvironments at different anatomic sites.

## Discussion

We have previously reported the presence of IgG4^+^ infiltrating cells in melanoma tumors and the functional contributions of IgG4 in promoting tumor progression by impairing immunity. In the same study, preliminary evaluations of circulating IgG4 in sera from 33 melanoma patients a correlation between higher IgG4 levels and less favorable patient prognosis.^22^ We therefore wished to elucidate whether antibody selection profiles featuring biased production of IgG4 may be associated with disease progression among patients with local or metastatic melanoma.

In a 167 patient cohort, we found higher overall circulating IgG4 levels as compared to those from 104 healthy volunteer sera. Consistent with our prior study and in concordance with published findings describing associations between inflammatory pathologies and malignant diseases, we confirm that elevated serum levels of IgG4 are negative predictors of progression-free and overall survival in patients with melanoma. Furthermore, our findings support the prognostic value of IgG4 in early stage (Stage I–II) melanoma, where an unmet need exists for predictors of disease progression.

Associations of elevated serum IgG4 levels with increased risk of disease progression at early stages are supported by an elevated circulating IgG4+ B cell subset that is also predictive of the risk of disease progression in early stages (Stage I–II). We speculate that temporal and memory IgG4-biased immunity may be early indicators of active immunosuppression and indicate worse clinical outcomes, consistent with previous evidence that IgG4 antibodies contribute to impairment of effective immune responses. Therefore, immunomonitoring of circulating memory class-switched IgG4^+^ B cells may complement temporal immune markers such as serum proteins, cytokines and autoantibodies. From a clinical perspective, predicting which patients originally diagnosed with localized melanoma will develop metastatic disease remains a clinical challenge. Novel indicators of disease progression are required to improve disease management, especially at early disease stages. Presently, invasive interventions such as sentinel lymph node biopsy (SNB) are used to elucidate disease stage, to predict the risk of progressive disease and identify treatment options. In the future, comparing the predictive value of SNB with serum readouts, such as circulating IgG4, may permit less intrusive clinical tools for patient management.

In our cohort, we confirm that the known serum biomarker LDH is an independent prognostic indicator in melanoma.^9,26^ However, elevated serum LDH levels may be associated with a range of non-neoplastic pathologies such as hemolysis, hepatitis, myocardial infarction, or infections, yielding false-positive results in melanoma.^26^ Elevated serum IgG4 levels are also described in autoimmune pancreatitis, indicating that IgG4 may also be influenced by biological mechanisms unrelated to malignancy,^27^ albeit only in a subset of patients. This limitation should be further investigated in future studies evaluating the clinical utility of serum IgG4 in melanoma.

Clinical practice has benefited by the companion diagnostic test for BRAF V600 mutations, used to select patients who may benefit from targeted pathway inhibitor drugs like vemurafenib.^28^ Based on emerging evidence that elements of the immune response play key roles in tumor surveillance and success of therapies,^29^ it is possible that components of the immune system may become the next important source of tissue prognostic indicators. Infiltrating immune cells, including various B cell subsets in melanoma ^30^ and other solid tumors, may be indicative of the strength and nature of tumor immune responses and could be linked to specific clinical outcomes. Here, we observe a prominent IgG4^+^ cell infiltrate in a proportion of melanomas across tumor locations, tumor thicknesses and stages of disease. Together with elevated IgG4 serum levels and increased circulating IgG4^+^ B cells, this may indicate that immunosuppressive mechanisms are present and active at both the peripheral and local levels throughout disease stages. Although it was not feasible here to associate these infiltrates with patient history or clinical outcomes, in future studies IgG4^+^ infiltrates may be examined along with immune infiltrates associated with tumor-induced suppressive mechanisms, such as Tregs.. Levels of IgG4^+^ cell infiltration may also be evaluated along with clinicopathological features such as ulceration and mitotic rate, or combined with other tissue markers such as S-100B.

Dysregulated antibody profiles are associated with a variety of inflammatory pathologies, including malignant melanoma and other types of cancer. The production of IgG4 subclass antibodies is promoted in IL-10-driven alternative T helper type 2 (Th2)-biased immune conditions and in response to prolonged antigen exposure. This results in class-switching and production of IgG4, a subclass known to trigger ineffective Fc-mediated cell activation due to distinctive conformation features.^31,32^ Melanoma tumor antigen-specific antibodies of the IgG4 isotype are less potent at engendering effector cell–mediated tumor cell killing *in vitro* and in restricting tumor growth *in vivo* as compared to IgG1. Importantly, both antigen-specific and nonspecific IgG4 antibodies can impair IgG1-mediated tumoricidal functions and this IgG4 blockade is mediated through interfering with IgG1 engaging activity at Fcγ receptors.^22,33^ Although tumor cell-reactive IgG4 antibodies have been detected both in patient circulation and in cutaneous melanoma lesions, Fc receptor binding and blockade of effector cell activation appears to form a key aspect of the immunomodulatory functions of IgG4. This Fc receptor blockade mechanism may operate in conjunction with recognition and occupancy of tumor antigens on the surface of cancer cells by IgG4, hindering engagement of potentially tumoricidal antibodies. Consistent with immune tolerance and inhibition of antitumor antibody functions described as features of IgG4, humoral immune profiles skewed toward IgG4 expression correlate with low protective efficacy of some HIV vaccination approaches.^34,35^ Immune bias toward IgG4 is also associated with disease pathology in IgG4-related inflammatory diseases and with immune suppression in cancers, such as pancreatic and squamous cell carcinomas and extrahepatic cholangiocarcinomas.^18-20,36^ Antibodies of the IgG subclass are also associated with patient responses to allergen immunotherapies.^37-39^ Manifestations of IgG4 humoral immune response bias include: 1) elevated or dysregulated IgG4 serum levels; 2) the presence of IgG4^+^ immune cell infiltrates in affected tissues; and 3) associations of tissue-resident IgG4^+^ cells with regulatory cells such as FoxP3^+^ Tregs. In concordance with previous reports, our data here provide rationale for further evaluation of dysregulated IgG4 antibody immunity as a prognostic indicator in melanoma, perhaps along with other immunoregulatory components. Clinical applications in regards to other malignant and inflammatory diseases or in monitoring host responses to vaccination and immunotherapies may also warrant future investigation.^40^ Various components of the humoral immune system, including circulating antibodies, have been widely studied in relation to autoimmune, inflammatory, allergic and malignant diseases. In order to assess early disease, circulating antibodies monitored via microarray-based technologies could complement other biomarkers, such as circulating antigens, perhaps combined using emerging and novel bioinformatics tools.^41-43^

Monitoring of dysregulated components of host immune surveillance, such as IgG4 or cell subsets that indicate immunosuppression,^44,45^ could help predict non-responders to immunotherapies including monoclonal antibodies. Simultaneous serological, diagnostic and immunological readouts may also be envisaged, based on the premise that multiple markers each with a unique sensitivity and specificity combined using an appropriate algorithm may harbor robust diagnostic or prognostic potential.^43^ In concordance, we found that combined analysis of serum LDH and IgG4 levels had improved predictive value for disease progression. Combined clinical tools, such as radiological assessments along with the clinically-used serum LDH at baseline, have been reported to improve predictions of patient outcomes following therapy with the monoclonal antibody bevacizumab.^46^ Thus, assessments of multiple parameters may provide better prognostic utility for patient management in the future. Larger clinical studies are required to identify and validate more reliable combinations of disease biomarkers, including IgG4 in melanoma, to derive improved models of personalized medicine.

The immunological mechanisms that govern the nature of humoral responses in cancer remain only partly elucidated, including those underlying biased IgG4 subclass expression in patient circulation and melanoma tumors. Although IgE and IgG4 are promoted by Th2 conditions, preferential expression of IL-10 in melanoma tumors could polarize a classical Th2-type immunity toward IgG4.^47,48^ At early phases of carcinogenesis, initial stress signals and release of mediators such as IL-25, TSLP and IL-33 by epithelial cells in the skin may drive IL-4 production and perhaps favor IgE which may confer protection.^49–51^ However, the persistent production of IL-10 by tumor cells and by tumor-promoted Tregs and macrophages may skew Th2 immunity in favor of IgG4 at early and late stages of disease manifestation. In this sense, higher than normal IgG4 levels particularly in early stage melanoma may signify active immunosuppressive forces already in operation.

In the future, it can be envisaged that antibodies less prone to IgG4 immune impairment, treatments that counteract IL-10-driven inflammation, or interventions that promote classical rather than alternative Th2 immunity could help refocus antitumoral immune signals. Strategies may include induction of protective Th2 responses that could perhaps drive *de novo* IgE or class switching from IgG4 to IgE.^52-56^ Alternatively, exploiting the potent functions of IgE class antibodies directed against tumor antigens may harness the intrinsic capacity of IgE for immune surveillance in Th2-biased tissue environments such as melanoma and other solid tumors.^57,58^ Taken together with emerging insights into the humoral arm of immunity in cancer, elucidating the significance and mechanisms of immunoglobulin subclasses may point to therapeutic strategies less prone to tumor-associated immune impairment.

In summary, we confirm that IgG4, a purported indicator of immunosuppressive forces in melanoma is elevated in the circulation of melanoma patients, is associated with worse clinical outcomes, and is a negative prognostic indicator at early stages of melanoma. Broader applications for IgG4 monitoring may be complimented by other clinical tools, such as LDH, or upon incorporation into immune profiling algorithms. Larger prospective studies will ascertain the clinical significance of IgG4, perhaps along with other immunoglobulin or immune cell signatures, in facilitating more accurate prediction of progressive disease. This may pave the way for improved patient stratification and optimal personalized therapies.

## Materials and Methods

### Sample collection

Human specimens were collected with written informed consent (approved by the Guy's Research Ethics Committee, Guy's and St. Thomas' NHS Trust, UK). Patients were staged and classified according to the American Joint Committee on Cancer Melanoma Staging and Classification criteria.^4^ Patients with Stage I–II melanoma had regional disease with no lymph node involvement or any other metastases; patients with Stage III melanoma had tumor cells detected in at least one lymph node; patients with Stage IV melanoma had distant metastases. Disease was staged by means of physical examinations by a trained dermatologist and verified by histopathological evaluations. The extent of disease was also assessed by means of appropriate clinical imaging tools such as PET-CT or CT. The study endpoints were progression-free survival and overall survival.

### IgG subclass and LDH analyses in human sera

Luminex bead array assay kits for assessments of IgG4 subclass antibodies in human sera were used according to the manufacturers' instructions. Data analyses to assess IgG subclass antibodies were conducted using Milliplex® MAP (Millipore, Cat.-No HGAMMAG-301K) and data were acquired and analyzed using FlexMap3D (Luminex Corporation). LDH serum levels were measured at the central laboratories of the associated hospital.

### Immunohistochemical evaluations

Tissue microarrays (TMA; BioMax, n = 256) of paraffin embedded sections were cut at 6–8 μm thickness on a microtome (Leica) and dried overnight at 60°C. Prior to staining, sections were deparaffinized in xylene for 20 min and rehydrated by serial incubations in alcohol. Heat-induced antigen retrieval was performed in a 95°C water bath using a citric acid solution (pH = 6.0). Sections were subsequently blocked with human FcR blocking reagent (Miltenyi Biotec; Cat-No: 130-059-90) according to the manufacturer's instructions and stained using a mouse anti-human IgG4 antibody (BD Biosciences; Cat-No:555881), followed by a rabbit anti-mouse IgG antibody conjugated to biotin (DAKO; Cat-No: E035401-2). IgG4^+^ cell infiltrates were detected using the VECTOR Red Alkaline Phosphatase Substrate Kit with levamisole (Vector Labs; Cat-No: SK-5100). All sections were mounted in DPX mounting solution and analyzed on a Zeiss Axiophot microscope using a 10x magnification lens (Carl Zeiss) and NIS-Elements imaging software (Nikon). Cell density of sections stained for IgG4^+^ B cell infiltration was evaluated by 2 independent researchers to identify the occupancy of infiltration per high power field (10x) using the following criteria for occupancy: “negative” = 0% infiltration; “low” < 25% infiltration; and “high” > 25% infiltration.

### Peripheral blood cell isolation and cytofluorimetric analysis of circulating IgG4^**+**^ B cells

Peripheral blood mononuclear cells (PBMC) were isolated from venous blood by density centrifugation over Ficoll-Paque Plus (GE Healthcare; Cat-No: 17144003). Cells were placed in RPMI 1640 medium (Life Technologies; Cat-No:31870-025) with 11.25% human serum albumin (Gemini Bio-Products, West Sacramento; Cat.-No: 800-125P), frozen in 10% DMSO (Sigma; Cat.-No: D2650) and stored in liquid nitrogen. Prior to analyses, cells were thawed and stained with fluorophore-conjugated antibodies against the following surface markers (BD Biosciences; Cat.-No: 560777, 562859, 562653, 641397,555947, 555881): CD45, CD22, CD19, CD3, CD14, IgG4. Prior to use, the anti-IgG4 antibody was pre-labeled with R-phycoerythrin (Innova Bioscience; Cat-No: 703-0030) following the manufacturer's instructions. Dead cells were excluded by staining with LIVE/DEAD® Fixable Dead Cell Stains (Life Technologies; L23105). Samples were subsequently acquired on a 5-laser SORP Fortessa (BD Bioscience) flow cytometer and data analyzed using the same instrument application settings across different experiments. IgG4^+^ cells were analyzed based on gating with an isotype control. All sample analyses were performed using FlowJo software (Treestar).

### Statistical analyses

Human serum and cell populations were assessed for normal Gaussian distribution with D'Agostino and Pearson omnibus normality test, followed by Kruskal–Wallis one-way analysis of variance with post-hoc Dunn's test. For comparisons between 2 groups and where populations were not normally distributed (analyzed by D'Agostino's K-square test for normality) statistical significance between groups was calculated using the Mann-Whitney-U-test. For correlation and patient survival analyses, Spearman and Mantel-Cox analyses were applied. Receiver Operating Characteristic (ROC) analyses and area under the curve (AUC) analyses were performed to assess the prognostic capacity of IgG4 in patient sera. The prognostic capacity of LDH and IgG4 combined serum tests was evaluated by applying a ROC analysis and calculating the area under the curve (AUC3). Threshold values were calculated via the Youden's Index^25^ to compare patient serum IgG4 with LDH and determine hazard ratios (HR) for the risk of progression free survival (PFS) and overall survival (OS). The cohort was analyzed using multivariate analysis. The maximum follow-up (from time of venipuncture to the last documented clinical visit) was 4.82 years with a median follow-up for overall survival of 2.08 years for LDH and 2.04 years for IgG4. The correlation between IgG4 and C-reactive protein, between IgG4 and white blood cell count and between IgG4 and breslow thickness were calculated using a Spearman correlation as the cohorts were not normally distributed.

Comparative IgG4 serum level analyses among melanoma patients with subsequent stable (SD) or progressive disease (PD) were conducted. Patients were segregated into: a) those with stable disease (neither progressive disease nor subsequent relapse during study period); and b) those with progressive disease (either active progressing disease monitored by imaging tools such as PET-CT, or subsequent relapse during study period). Patients could only be up-staged from their initial staging during the study period.

Error bars in all figures represent standard error of mean (SEM). All statistical tests were performed using GraphPad Prism Version 6, with the exception of the calculation of hazard ratio (HR), which was performed using the SAS software version 9.2 (SAS Institute Inc.). All reported *P* values are derived from 2-sided comparisons with values of less than 0.05 considered to indicate statistical significance.

## Supplemental Material

Supp. Figure 1 and Table 1

## References

[1] Chapman PB, Hauschild A, Robert C, Haanen JB, Ascierto P, Larkin J, Dummer R, Garbe C, Testori A, Maio M, et al. Improved survival with vemurafenib in melanoma with BRAF V600E mutation. N Engl J Med 2011; 364:2507-16; PMID:21639808; http://dx.doi.org/10.1056/NEJMoa1103782 21639808 PMC3549296

[2] Hodi FS, O'Day SJ, McDermott DF, Weber RW, Sosman JA, Haanen JB, Gonzalez R, Robert C, Schadendorf D, Hassel JC, et al. Improved survival with ipilimumab in patients with metastatic melanoma. N Engl JMed 2010; 363:711-23; PMID:20525992; http://dx.doi.org/10.1056/NEJMoa1003466 20525992 PMC3549297

[3] Corrie PG, Marshall A, Dunn JA, Middleton MR, Nathan PD, Gore M, Davidson N, Nicholson S, Kelly CG, Marples M, et al. Adjuvant bevacizumab in patients with melanoma at high risk of recurrence (AVAST-M): preplanned interim results from a multicentre, open-label, randomised controlled phase 3 study. Lancet Oncol 2014; 15:620-30; PMID:24745696; http://dx.doi.org/10.1016/S1470-2045(14)70110-X 24745696

[4] Balch CM, Gershenwald JE, Soong SJ, Thompson JF, Atkins MB, Byrd DR, Buzaid AC, Cochran AJ, Coit DG, Ding S, et al. Final version of 2009 AJCC melanoma staging and classification. J Clin Oncol 2009; 27:6199-206; PMID:19917835; http://dx.doi.org/10.1200/JCO.2009.23.4799 19917835 PMC2793035

[5] de Vries M, Speijers MJ, Bastiaannet E, Plukker JT, Brouwers AH, van Ginkel RJ, Suurmeijer AJ, Hoekstra HJ. Long-term follow-up reveals that ulceration and sentinel lymph node status are the strongest predictors for survival in patients with primary cutaneous melanoma. Eur J Surg Oncol 2011; 37:681-7; PMID:21636244; http://dx.doi.org/10.1016/j.ejso.2011.05.003 21636244

[6] Morton DL, Thompson JF, Cochran AJ, Mozzillo N, Elashoff R, Essner R, Nieweg OE, Roses DF, Hoekstra HJ, Karakousis CP, et al. Sentinel-node biopsy or nodal observation in melanoma. N Engl J Med 2006; 355:1307-17; PMID:17005948; http://dx.doi.org/10.1056/NEJMoa060992 17005948

[7] de Vries M, Vonkeman WG, van Ginkel RJ, Hoekstra HJ. Morbidity after inguinal sentinel lymph node biopsy and completion lymph node dissection in patients with cutaneous melanoma. Eur J Surg Oncol 2006; 32:785-9; PMID:16806794; http://dx.doi.org/10.1016/j.ejso.2006.05.003 16806794

[8] Morton DL, Thompson JF, Cochran AJ, Mozzillo N, Nieweg OE, Roses DF, Hoekstra HJ, Karakousis CP, Puleo CA, Coventry BJ, et al. Final trial report of sentinel-node biopsy versus nodal observation in melanoma. N Engl J Med 2014; 370:599-609; PMID:24521106; http://dx.doi.org/10.1056/NEJMoa1310460 24521106 PMC4058881

[9] Poo-Hwu WJ, Ariyan S, Lamb L, Papac R, Zelterman D, Hu GL, Brown J, Fischer D, Bolognia J, Buzaid AC. Follow-up recommendations for patients with American Joint Committee on Cancer Stages I–III malignant melanoma. Cancer 1999; 86:2252-8; PMID:10590365; http://dx.doi.org/10.1002/(SICI)1097-0142(19991201)86:11%3c2252::AID-CNCR12%3e3.0.CO;2-Q 10590365

[10] Kelderman S, Heemskerk B, van Tinteren H, van den Brom RR, Hospers GA, van den Eertwegh AJ, Kapiteijn EW, de Groot JW, Soetekouw P, Jansen RL, et al. Lactate dehydrogenase as a selection criterion for ipilimumab treatment in metastatic melanoma. Cancer Immunol, Immunother 2014; 63:449-58; PMID:24609989 24609989 10.1007/s00262-014-1528-9 PMC11029318

[11] Tandler N, Mosch B, Pietzsch J. Protein and non-protein biomarkers in melanoma: a critical update. Amino acids 2012; 43:2203-30; PMID:23053020; http://dx.doi.org/10.1007/s00726-012-1409-5 23053020

[12] Karakousis G, Yang R, Xu X. Circulating melanoma cells as a predictive biomarker. J Invest Dermatol 2013; 133:1460-2; PMID:23673501; http://dx.doi.org/10.1038/jid.2013.34 23673501

[13] Khoja L, Lorigan P, Zhou C, Lancashire M, Booth J, Cummings J, Califano R, Clack G, Hughes A, Dive C. Biomarker utility of circulating tumor cells in metastatic cutaneous melanoma. J Invest Dermatol 2013; 133:1582-90; PMID:23223143; http://dx.doi.org/10.1038/jid.2012.468 23223143

[14] Khoja L, Lorigan P, Dive C, Keilholz U, Fusi A Circulating tumor cells as tumor biomarkers in melanoma: detection methods and clinical relevance. Ann Oncol 2015; 26(1):33-9; PMID:24907634; http://dx.doi.org/0.1093/annonc/mdu207 24907634 10.1093/annonc/mdu207

[15] Karagiannis P, Fittall M, Karagiannis SN. Evaluating biomarkers in melanoma. Front Oncol 2014; 4:383; PMID:25667918; http://dx.doi.org/10.3389/fonc.2014.00383 25667918 PMC4304353

[16] Restrepo L, Stafford P, Johnston SA. Feasibility of an early Alzheimer's disease immunosignature diagnostic test. J Neuroimmunol 2013; 254:154-60; PMID:23084373; http://dx.doi.org/10.1016/j.jneuroim.2012.09.014 23084373

[17] Hughes AK, Cichacz Z, Scheck A, Coons SW, Johnston SA, Stafford P. Immunosignaturing can detect products from molecular markers in brain cancer. PloS one 2012; 7:e40201; PMID:22815729; http://dx.doi.org/10.1371/journal.pone.0040201 22815729 PMC3397978

[18] Daveau M, Pavie-Fischer J, Rivat L, Rivat C, Ropartz C, Peter HH, Cesarini JP, Kourilsky FM. IgG4 subclass in malignant melanoma. J Natl Cancer Inst 1977; 58:189-92; PMID:833869 833869 10.1093/jnci/58.2.189

[19] Harada K, Shimoda S, Kimura Y, Sato Y, Ikeda H, Igarashi S, Ren XS, Sato H, Nakanuma Y. Significance of immunoglobulin G4 (IgG4)-positive cells in extrahepatic cholangiocarcinoma: molecular mechanism of IgG4 reaction in cancer tissue. Hepatology 2012; 56:157-64; PMID:22290731; http://dx.doi.org/10.1002/hep.25627 22290731

[20] Vaglio A, Strehl JD, Manger B, Maritati F, Alberici F, Beyer C, Rech J, Sinico RA, Bonatti F, Battistelli L, et al. IgG4 immune response in Churg-Strauss syndrome. Ann Rheum Dis 2012; 71:390-3; PMID:22121132; http://dx.doi.org/10.1136/ard.2011.155382 22121132

[21] Gilbert AE, Karagiannis P, Dodev T, Koers A, Lacy K, Josephs DH, Takhar P, Geh JL, Healy C, Harries M, et al. Monitoring the systemic human memory B cell compartment of melanoma patients for anti-tumor IgG antibodies. PloS one 2011; 6:e19330; PMID:21559411; http://dx.doi.org/10.1371/journal.pone.0019330 21559411 PMC3084832

[22] Karagiannis P, Gilbert AE, Josephs DH, Ali N, Dodev T, Saul L, Correa I, Roberts L, Beddowes E, Koers A, et al. IgG4 subclass antibodies impair antitumor immunity in melanoma. J Clin Invest 2013; 123:17; http://dx.doi.org/10.1172/JCI65579 PMC3613918 23454746

[23] Karagiannis P, Gilbert AE, Nestle FO, Karagiannis SN. IgG4 antibodies and cancer-associated inflammation: Insights into a novel mechanism of immune escape. Oncoimmunology 2013; 2:e24889; PMID:24073371; http://dx.doi.org/10.4161/onci.24889 24073371 PMC3782134

[24] Baker SG. The central role of receiver operating characteristic (ROC) curves in evaluating tests for the early detection of cancer. J Natl Cancer Inst 2003; 95:511-5; PMID:12671018; http://dx.doi.org/10.1093/jnci/95.7.511 12671018

[25] Youden WJ. Index for rating diagnostic tests. Cancer 1950; 3:32-5; PMID:15405679; http://dx.doi.org/10.1002/1097-0142(1950)3:1%3c32::AID-CNCR2820030106%3e3.0.CO;2-3 15405679

[26] Vereecken P, Cornelis F, Van Baren N, Vandersleyen V, Baurain JF. A synopsis of serum biomarkers in cutaneous melanoma patients. Dermatol Res Pract 2012; 2012:260643; PMID:22287956; http://dx.doi.org/10.1155/2012/260643 22287956 PMC3263591

[27] Kawa S, Ito T, Watanabe T, Maruyama M, Hamano H, Muraki T, Arakura N. The Utility of Serum IgG4 Concentrations as a Biomarker. Int J Rheumatol 2012; 2012:198314; PMID:22536256; http://dx.doi.org/10.1155/2012/198314 22536256 PMC3321274

[28] Halait H, Demartin K, Shah S, Soviero S, Langland R, Cheng S, Hillman G, Wu L, Lawrence HJ. Analytical performance of a real-time PCR-based assay for V600 mutations in the BRAF gene, used as the companion diagnostic test for the novel BRAF inhibitor vemurafenib in metastatic melanoma. Diagn Mol Pathol 2012; 21:1-8; PMID:22306669; http://dx.doi.org/10.1097/PDM.0b013e31823b216f 22306669

[29] Ilieva KM, Correa I, Josephs DH, Karagiannis P, Egbuniwe IU, Cafferkey MJ, Spicer JF, Harries M, Nestle FO, Lacy KE, et al. Effects of BRAF mutations and BRAF inhibition on immune responses to melanoma. Mol Cancer Ther 2014; 13:2769-83; PMID:25385327; http://dx.doi.org/10.1158/1535-7163.MCT-14-0290 25385327 PMC4258403

[30] Egbuniwe IU, Karagiannis SN, Nestle FO, Lacy KE. Revisiting the role of B cells in skin immune surveillance. Trends Immunol 2015; 36:102-11; PMID:25616715; http://dx.doi.org/10.1016/j.it.2014.12.006 25616715

[31] Lighaam LC, Vermeulen E, Bleker T, Meijlink KJ, Aalberse RC, Barnes E, Culver EL, van Ham SM, Rispens T. Phenotypic differences between IgG4+ and IgG1+ B cells point to distinct regulation of the IgG4 response. J Allergy Clin Immunol 2014; 133:267-70 e1–6; PMID:24074895; http://dx.doi.org/10.1016/j.jaci.2013.07.044 24074895

[32] Davies AM, Rispens T, Ooijevaar-de Heer P, Gould HJ, Jefferis R, Aalberse RC, Sutton BJ. Structural determinants of unique properties of human IgG4-Fc. J Mol Biol 2014; 426:630-44; PMID:24211234; http://dx.doi.org/10.1016/j.jmb.2013.10.039 24211234 PMC3905167

[33] Karagiannis P, Gilbert AE, Josephs DH, Ali N, Dodev T, Saul L, Correa I, Roberts L, Beddowes E, Koers A, et al. IgG4 subclass antibodies impair antitumor immunity in melanoma. J Clin Invest 2013; 123:1457-74; PMID:23454746; http://dx.doi.org/10.1172/JCI65579 23454746 PMC3613918

[34] Chung AW, Ghebremichael M, Robinson H, Brown E, Choi I, Lane S, Dugast AS, Schoen MK, Rolland M, Suscovich TJ, et al. Polyfunctional Fc-effector profiles mediated by IgG subclass selection distinguish RV144 and VAX003 vaccines. Sci Transl Med 2013; 6:228ra38 10.1126/scitranslmed.3007736 24648341

[35] Lai JI, Licht AF, Dugast AS, Suscovich T, Choi I, Bailey-Kellogg C, Alter G, Ackerman ME. Divergent antibody subclass and specificity profiles but not protective HLA-B alleles are associated with variable antibody effector function among HIV-1 controllers. J Virol 2014; 88:2799-809; PMID:24352471; http://dx.doi.org/10.1128/JVI.03130-13 24352471 PMC3958053

[36] Okazaki K, Uchida K, Koyabu M, Miyoshi H, Ikeura T, Takaoka M. IgG4 Cholangiopathy - current concept, diagnosis and pathogenesis. J Hepatol 2014; 61(3):690-5; PMID:24768756 24768756 10.1016/j.jhep.2014.04.016

[37] Keet CA, Frischmeyer-Guerrerio PA, Thyagarajan A, Schroeder JT, Hamilton RG, Boden S, Steele P, Driggers S, Burks AW, Wood RA The safety and efficacy of sublingual and oral immunotherapy for milk allergy. J Allergy Clin Immunol 2012; 129:448-55, 55 e1–5 22130425 10.1016/j.jaci.2011.10.023 PMC3437605

[38] James LK, Shamji MH, Walker SM, Wilson DR, Wachholz PA, Francis JN, Jacobson MR, Kimber I, Till SJ, Durham SR. Long-term tolerance after allergen immunotherapy is accompanied by selective persistence of blocking antibodies. J Allergy Clin Immunol 2011; 127:509-16 e1–5; PMID:21281875; http://dx.doi.org/10.1016/j.jaci.2010.12.1080 21281875

[39] Francis JN, James LK, Paraskevopoulos G, Wong C, Calderon MA, Durham SR, Till SJ. Grass pollen immunotherapy: IL-10 induction and suppression of late responses precedes IgG4 inhibitory antibody activity. J Allergy Clin Immunol 2008; 121:1120-5 e2; PMID:18374405; http://dx.doi.org/10.1016/j.jaci.2008.01.072 18374405

[40] Saul L, Josephs DH, Cutler K, Bradwell A, Karagiannis P, Selkirk C, Gould HJ, Jones P, Spicer JF, Karagiannis SN. Comparative reactivity of human IgE to cynomolgus monkey and human effector cells and effects on IgE effector cell potency. mAbs 2014; 6:509-22; PMID:24492303; http://dx.doi.org/10.4161/mabs.27828 24492303 PMC3984339

[41] Maecker HT, Nolan GP, Fathman CG. New technologies for autoimmune disease monitoring. Curr Opin Endocrinol Diabetes Obes 2010; 17:322-8; PMID:20531181; http://dx.doi.org/10.1097/MED.0b013e32833ada91 20531181 PMC4311749

[42] Sykes KF, Legutki JB, Stafford P. Immunosignaturing: a critical review. Trends Biotechnol 2013; 31:45-51; PMID:23219199; http://dx.doi.org/10.1016/j.tibtech.2012.10.012 23219199

[43] Tsoka S, Ainali C, Karagiannis P, Josephs DH, Saul L, Nestle FO, Karagiannis SN. Toward prediction of immune mechanisms and design of immunotherapies in melanoma. Crit Rev Biomed Eng 2012; 40:279-94; PMID:23140120; http://dx.doi.org/10.1615/CritRevBiomedEng.v40.i4.40 23140120

[44] Martens A, Zelba H, Garbe C, Pawelec G, Weide B. Monocytic myeloid-derived suppressor cells in advanced melanoma patients: Indirect impact on prognosis through inhibition of tumor-specific T-cell responses? Oncoimmunology 2014; 3:e27845; PMID:24800171; http://dx.doi.org/10.4161/onci.27845 24800171 PMC4008451

[45] Weide B, Martens A, Zelba H, Stutz C, Derhovanessian E, Di Giacomo AM, Maio M, Sucker A, Schilling B, Schadendorf D, et al. Myeloid-derived suppressor cells predict survival of patients with advanced melanoma: comparison with regulatory T cells and NY-ESO-1- or melan-A-specific T cells. Clin Cancer Res 2014; 20:1601-9; PMID:24323899; http://dx.doi.org/10.1158/1078-0432.CCR-13-2508 24323899

[46] Gray MR, Martin del Campo S, Zhang X, Zhang H, Souza FF, Carson WE 3rd, Smith AD. Metastatic melanoma: lactate dehydrogenase levels and CT imaging findings of tumor devascularization allow accurate prediction of survival in patients treated with bevacizumab. Radiology 2014; 270:425-34; PMID:24072776; http://dx.doi.org/10.1148/radiol.13130776 24072776 PMC3985552

[47] Jeannin P, Lecoanet S, Delneste Y, Gauchat JF, Bonnefoy JY. IgE versus IgG4 production can be differentially regulated by IL-10. J Immunol 1998; 160:3555-61; PMID:9531318 9531318

[48] Punnonen J, de Waal Malefyt R, van Vlasselaer P, Gauchat JF, de Vries JE. IL-10 and viral IL-10 prevent IL-4-induced IgE synthesis by inhibiting the accessory cell function of monocytes. J Immunol 1993; 151:1280-9; PMID:8393044 8393044

[49] Komai-Koma M, Brombacher F, Pushparaj PN, Arendse B, McSharry C, Alexander J, Chaudhuri R, Thomson NC, McKenzie AN, McInnes I, et al. Interleukin-33 amplifies IgE synthesis and triggers mast cell degranulation via interleukin-4 in naive mice. Allergy 2012; 67:1118-26; PMID:22702477; http://dx.doi.org/10.1111/j.1398-9995.2012.02859.x 22702477 PMC3660789

[50] Strid J, Sobolev O, Zafirova B, Polic B, Hayday A. The intraepithelial T cell response to NKG2D-ligands links lymphoid stress surveillance to atopy. Science 2011; 334:1293-7; PMID:22144628; http://dx.doi.org/10.1126/science.1211250 22144628 PMC3842529

[51] Cipolat S, Hoste E, Natsuga K, Quist SR, Watt FM. Epidermal barrier defects link atopic dermatitis with altered skin cancer susceptibility. eLife 2014; 3:e01888; PMID:24843010; http://dx.doi.org/10.7554/eLife.01888 24843010 PMC4007207

[52] Singer J, Jensen-Jarolim E. IgE-based immunotherapy of cancer: challenges and chances. Allergy 2014; 69:137-49; PMID:24117861; http://dx.doi.org/10.1111/all.12276 24117861 PMC4022995

[53] Jensen-Jarolim E, Achatz G, Turner MC, Karagiannis S, Legrand F, Capron M, Penichet ML, Rodríguez JA, Siccardi AG, Vangelista L, et al. AllergoOncology: the role of IgE-mediated allergy in cancer. Allergy 2008; 63:1255-66; PMID:18671772; http://dx.doi.org/10.1111/j.1398-9995.2008.01768.x 18671772 PMC2999743

[54] Nigro EA, Soprana E, Brini AT, Ambrosi A, Yenagi VA, Dombrowicz D, Siccardi AG, Vangelista L. An antitumor cellular vaccine based on a mini-membrane IgE. J Immunol 2012; 188:103-10; PMID:22124126; http://dx.doi.org/10.4049/jimmunol.1101842 22124126

[55] Riemer AB, Untersmayr E, Knittelfelder R, Duschl A, Pehamberger H, Zielinski CC, Scheiner O, Jensen-Jarolim E. Active induction of tumor-specific IgE antibodies by oral mimotope vaccination. Cancer Res 2007; 67:3406-11; PMID:17409451; http://dx.doi.org/10.1158/0008-5472.CAN-06-3758 17409451

[56] Dalessandri T, Strid J. Beneficial autoimmunity at body surfaces - immune surveillance and rapid type 2 immunity regulate tissue homeostasis and cancer. Front Immunol 2014; 5:347; PMID:25101088; http://dx.doi.org/10.3389/fimmu.2014.00347 25101088 PMC4105846

[57] Josephs DH, Spicer JF, Karagiannis P, Gould HJ, Karagiannis SN. IgE immunotherapy: a novel concept with promise for the treatment of cancer. mAbs 2014; 6:54-72; PMID:24423620; http://dx.doi.org/10.4161/mabs.27029 24423620 PMC3929456

[58] Josephs DH, Bax HJ, Karagiannis SN. Tumour-associated macrophage polarisation and re-education with immunotherapy. Front Biosci (Elite Ed) 2015; 7:293-308; PMID:25553381 25553381 10.2741/E735

