# Supplementary material for: Elevated IgG4 in patient circulation is associated with the risk of disease progression in melanoma
Source: Oncoimmunology. 2015 Jun 3;4(11):e1032492. doi: 10.1080/2162402X.2015.1032492 (PMC4590000; doi:10.1080/2162402X.2015.1032492)
Supplement: Supp. Figure 1 and Table 1 [file koni-04-11-1032492-s001.zip › Supplementary Table 1.docx]

**Supplementary Table 1**

SD: standard deviation

NA: not applicable
